# EST and microarray analysis of horn development in *Onthophagus *beetles

**DOI:** 10.1186/1471-2164-10-504

**Published:** 2009-10-30

**Authors:** Teiya Kijimoto, James Costello, Zuojian Tang, Armin P Moczek, Justen Andrews

**Affiliations:** 1 Department of Biology, Indiana University, Bloomington, Indiana, 47405, USA; 2 School of Informatics, Indiana University, Bloomington, Indiana, 47405, USA; 3 Center for Genomics and Bioinformatics, Indiana University, Bloomington, Indiana, 47405, USA

## Abstract

**Background:**

The origin of novel traits and their subsequent diversification represent central themes in evo-devo and evolutionary ecology. Here we explore the genetic and genomic basis of a class of traits that is both novel and highly diverse, in a group of organisms that is ecologically complex and experimentally tractable: horned beetles.

**Results:**

We developed two high quality, normalized cDNA libraries for larval and pupal *Onthophagus taurus *and sequenced 3,488 ESTs that assembled into 451 contigs and 2,330 singletons. We present the annotation and a comparative analysis of the conservation of the sequences. Microarrays developed from the combined libraries were then used to contrast the transcriptome of developing primordia of head horns, prothoracic horns, and legs. Our experiments identify a first comprehensive list of candidate genes for the evolution and diversification of beetle horns. We find that developing horns and legs show many similarities as well as important differences in their transcription profiles, suggesting that the origin of horns was mediated partly, but not entirely, by the recruitment of genes involved in the formation of more traditional appendages such as legs. Furthermore, we find that horns developing from the head and prothorax differ in their transcription profiles to a degree that suggests that head and prothoracic horns are not serial homologs, but instead may have evolved independently from each other.

**Conclusion:**

We have laid the foundation for a systematic analysis of the genetic basis of horned beetle development and diversification with the potential to contribute significantly to several major frontiers in evolutionary developmental biology.

## Background

The origin of novel traits and their subsequent diversification have been central themes in evolutionary biology ever since the discipline's inception over 150 years ago [1,2]. Specifically, the genetic, developmental, and ecological mechanisms, and the interactions between them, that allow novel phenotypes and functions to arise from pre-existing variation, continue to represent major frontiers in our understanding of phenotypic diversity. With the advent of modern -*omics *approaches, researchers have increasingly departed from a candidate gene or pathway approach and begun to explore organismal development and evolution from a genome, transcriptome, or proteome perspective, focusing in large part on existing genetic model systems such as *Drosophila *or *Caenorhabditis*. However, many key questions in evolutionary biology, including the mechanisms underlying organismal innovation, the role of plasticity in diversification, and the interplay between ecology and developmental evolution, are often difficult to address solely within the confines of classic model systems. Recent efforts have therefore begun to generate genomic and developmental genetic resources for organisms with promise as future model systems in evolutionary developmental biology and ecological genetics (e.g. butterflies: [3,4]; honey bees: reviewed in [5]; red flour beetle: [6]). Here we present and apply the first genomic resources to advance the study of a class of traits that is both novel and highly diverse in a group of organisms that is ecologically complex and experimentally tractable: beetle horns and horned beetles.

Beetle horns possess many characteristics that make them interesting models for integrating genetic, developmental, and environmental perspectives on the development and evolution of complex, novel traits (reviewed in [7]). First, beetle horns are major structures, often dominating the phenotype of their bearers. Second, beetle horns function as weapons of sexual selection, thus playing a major role in the behavioral ecology of individuals and populations. Third, beetle horns are inordinately variable, both within and between species, including differences in number, size, shape, and location. Moreover, diversity in horn expression is paralleled by amazing species richness. For instance, the genus *Onthophagus *currently contains over 2,400 extant species, making it the most speciose genus in the animal kingdom [8,9]. Forth, beetle horns are influenced in their expression by both genetic and environmental factors, ranging from absence of environmental sensitivity to complete determination by nutritional condition. In some cases, both extremes of environmental sensitivity can be found in different horn types expressed by the same individual [10]. Finally, beetle horns lack any obvious homology to structures in other insects or non-insect arthropods. Beetle horns are not modified antennae or mouthparts, but instead horns were "invented" by beetles in addition to their traditional appendages [11], and now provide their bearers with an important new function: a weapon used in male-male competition. Beetle horns and horned beetles therefore offer rich opportunities to explore the mechanisms of organismal innovation and diversification.

Beetle horns are rigid outgrowths of the exoskeleton that originate as epidermal outbuddings of the head or prothoracic epithelium. Horns lack joints, muscles, and nervous tissue. Several recent studies have begun to shed light on how beetle horns develop and differentiate during ontogeny [11-15] and showed that two developmentally dissociated processes contribute to the final degree of horn expression seen in adults: a prepupal growth phase late in larval development followed by a pupal remodeling phase just prior to the final adult molt (reviewed in [10]). As such, the development of horns shows many qualitative similarities to the development of traditional appendages, but also exhibits important differences. For instance, prothoracic horn primordia are frequently resorbed during the pupal stage in a sex- and species-specific manner, a phenomenon not usually associated with regular appendages [14]. Furthermore, earlier studies have also begun to question whether horns that develop in different body regions, such as the head *vs*. prothorax, constitute serial homologs, or instead may have evolved and diversified independently of each other [13,16].

Here we present the first steps toward a systematic analysis of the genetic and genomic basis of horn development and diversification in the genus *Onthophagus*. We first present the results of a comprehensive EST analysis of two normalized cDNA libraries obtained from two disparate developmental stages of *Onthophagus taurus*: larva and pupa. Second, using microarrays developed from our EST libraries we contrast the transcription profiles of the primordia of developing prothoracic horns, head horns, and legs right after the transition from larva to pupa. We then use these contrasts to identify candidate genes involved in the development and diversification of beetle horns. Furthermore, we examine two basic questions regarding the origin and diversification of horns. (a) Are horns highly simplified versions of more traditional appendages such as legs? If so, transcription profiles of developing horn primordia should largely match those of developing legs. If not, transcription profiles of developing horn primordia should only partly match those of developing legs and also include horn specific transcription signatures. (b) Are different horn types produced in different body regions homonomous, i.e. serial homologs of the same ancestral structure? If so, different horn types should exhibit highly similar transcription profiles. However, if different horn types originated and diversified independently of each other, transcription profiles may be predicted to exhibit important horn-type specific differences. We discuss the significance of our findings in the context of the biology of horned beetles in particular, and the origins and diversification of novel traits in general.

## Results

### Production and analysis of EST sequences

We constructed two normalized, size selected, and directionally cloned cDNA libraries from (i) heads and thoraces dissected from larvae and prepupae (referred to as *OtL *[larval]) and (ii) whole pupae (referred to as *OtP *[pupal]). Individual 5'- sequencing reads were generated from 3,756 randomly selected cDNA clones (1,874 larval and 1,882 pupal). The set of EST sequences were trimmed of vector sequence, adaptor sequence, and poly(A) tails, and filtered to remove sequences that were either low quality, chimeric, or shorter than 100 nucleotides (Methods). This yielded 3,488 high-quality sequences (1,783 larval and 1,705 pupal, Table 1) that are available at GenBank (accession numbers FG539013-FG542500). We then used ESTPiper [17] to assemble these sequences into contigs (see Methods). A total of 1,158 of these sequences were assembled into 451 contigs with an average of 2.6 clones per contig and a maximum of 11 clones per contig. The remaining 2,330 sequences did not assemble into contigs and are referred to as singletons. Thus, the 3,488 sequences collapse into 2,781 distinct sequences (451 contigs and 2,330 singletons) that we refer to as "non-redundant" sequences.

**Table 1:** Summary of cDNA libraries and EST sequence analysis

	**Larvae** **(OtL)**	**Pupae** **(OtP)**	**Mixed** **(OtL & OtP)**
median cDNA fragment size (nt)	715	975	-
average read length (nt)	624	667	645
raw reads	1,874	1,882	3,756
cleaned reads	1,783	1,705	3,488
assembled contigs	217	171	451
singletons	1,252	1,284	2,330
independent sequences	1,469	1,455	2,781
annotated	1,104	998	1,984

It is likely that some of the non-redundant sequences derive from the same transcript but do not overlap, possibly due to 5'-truncated cDNA clones. In order to estimate the magnitude of this redundancy, we aligned the *Onthophagus *non-redundant sequences to *Drosophila *proteins, filtered the alignments for highly similar matches (BLASTx, E-value < 10^-60^), and then manually examined the alignments for separate *Onthophagus *sequences that align to distinct regions of the same *Drosophila *protein. Among 534 non-redundant *Onthophagus *sequences we found 35 pairs of sequences that aligned to the same *Drosophila *protein. Of these, 19 pairs aligned with highly similar matches to different regions of the same protein, indicating they derive from non-overlapping regions of the same transcript; 12 pairs had co-linear alignments with 95-98% sequence identities, suggesting that they either derive from the same gene with polymorphisms and/or sequencing errors, or derive from highly similar duplicate genes; and 4 pairs appear to be splice variants. Thus, this sample of 534 *Onthophagus *non-redundant sequences represents approximately 499 distinct genes (93% unique). While this is not a random sample and thus can't be extrapolated to full set of non-redundant sequences, it does however indicate that false-negative assemblies are not a pervasive problem among the non-redundant sequences.

### Functional annotation of assembled sequences

Given that insects express a broad diversity of genes during metamorphosis [18], we expected that the larval and pupal ESTs would be a rich source of gene discovery. In order to provide a first pass annotation for the putative function of the *Onthophagus *gene sequences, we annotated the non-redundant sequences using the UniProtKB/TrEMBL protein sequence database (E-value < 10^-5^). This successfully annotated 71.3% of the non-redundant sequences. As expected, these annotations covered a wide diversity of biological and molecular functions including the major expected categories such as cellular processes, metabolic processes, biological regulation, multicellular organismal processes, and developmental processes (see Additional files 1 and 2). This, coupled with the low redundancy within the *Onthophagus *libraries, indicates the set of ESTs as a rich source for gene discovery.

Given that the cDNA libraries derive from animals undergoing metamorphosis, which involves dramatic remodeling of the insect body accompanied by complex patterns of gene expression, it was expected that the EST libraries would include genes involved in a wide range of developmental processes. Indeed the assembled sequences included over 75 genes with close sequence similarity to genes with important functions in the development of other arthropods (Table 2). Briefly, these included the following major groups: 1) genes involved in axis-specification, patterning and morphogenesis, including many transcription factors (*homothorax, extradenticle, spalt-related, bicaudal, prothoraxless, teashirt-like, Sex comb on midleg, Cephalothorax, Ultrabithorax, cut, tailup, pointed, Abdominal B, hairy, bab2, Additional sex combs*); 2) proteins involved in several signaling pathways, including MAPK pathways (*Epidermal growth factor-like protein, Star, MAP kinase-interacting serine/threonine kinase, licorne, puckered, DRas2, misshapen, discs large 1*) the Wnt receptor signaling pathway (*frizzled 4, shaggy, armadillo, hyrax, Wnt oncogene anolog 2*), the Notch signaling pathway (*Notch, fringe, dishevelled, kuzbanian, Enhancer of split, strawberry notch*), the Hedgehog signaling pathway (*hedgehog*), the TGF-beta related pathway (*bambi*, *cornichon*), and the Toll signaling pathway (*Spatzle-Processing Enzyme, pipe*); 3) genes involved in endocrine regulation of development including ecdysone signaling (*ultraspiracle, Ecdysone receptor, shade, disembodied, broad, Ecdysone-induced protein 78C, Ecdysone-induced protein 75B*) and juvenile hormone signaling (*Juvenile hormone epoxide hydrolase 3, Juvenile hormone acid methyl transferase*). These clones represent a rich set of annotated genes for future studies investigating the function of the respective pathways in *Onthophagus *development and evolution.

**Table 2:** Putative *Onthophagus taurus *orthologs with known functions in insect development and physiology

**Ot library ID**	**UniProtKB/FlyBase ID**	**Gene description**	**Identity (%)**	**E-value**
contig20^1^	FBpp0089324	shade	47.8	2.00E-44
contig47^3^	FBpp0077925	fringe	45.4	9.00E-60
contig54^1^	FBpp0088364	Autophagy-specific gene 12	53.2	7.00E-21
contig107^1^	FBpp0089159	shaggy	83.9	2.00E-49
contig122^3^	Q70WC9_TRICA	Homothorax	93.3	6.00E-10
contig146^1^	FBpp0076186	SHC-adaptor protein	48.4	4.00E-55
contig158^3^	FBpp0088946	transformer2	51.9	5.00E-22
contig187^3^	FBpp0075238	PDCD-5	57.8	1.00E-37
contig229^1^	Q17P53_AEDAE	Sptzle 1B (Spz1B)	28.5	2.00E-07
contig254^2^	Q70WD0_TRICA	Extradenticle	95.7	4.00E-91
contig255^2^	FBpp0081258	doublesex	81.8	5.00E-23
contig297^2^	Q1HTM7_9MYRI	Daughterless (Fragment)	32.9	2.00E-16
contig305^2^	FBpp0077659	Star	34.6	3.00E-47
contig356^3^	FBpp0089196	chickadee	81.7	4.00E-58
contig373^2^	FBpp0099954	held out wings	88.7	2.00E-78
contig404^3^	Q0IFK2_AEDAE	Map kinase-interacting serine/threonine kinase	87.3	8.00E-48
contig448^3^	Q176R2_AEDAE	PIWI	35.2	1.00E-57
OtL001A07	Q6RG14_AEDAE	Broad complex isoform Z1	95.1	4.00E-62
OtL001C11	Q178N5_AEDAE	Programmed cell death	59.6	8.00E-10
OtL003H10	Q68QF3_LITFO	Notch (Fragment)	61.8	5.00E-20
OtL004F07	FBpp0075677	Autophagy-specific gene 1	70.6	5.00E-09
OtL004G01	FBpp0089035	armadillo	87.7	1.00E-124
OtL005C09	FBpp0079823	spalt-related	54.3	3.00E-14
OtL005D10	Q206L4_AEDAE	Juvenile hormone acid methyl transferase	28.5	2.00E-13
OtL006H06	Q1HAY7_HOLDI	Epidermal growth factor-like protein	47.0	1.00E-64
OtL007E09	FBpp0073551	licorne	70.7	4.00E-76
OtL008H03	FBpp0070977	frizzled 4	50.3	7.00E-50
OtL011A08	HYEP1_CTEFE	Juvenile hormone epoxide hydrolase 1 (EC 3329)	48.5	2.00E-39
OtL011C06	CNI_DROME	Protein cornichon	75.9	3.00E-50
OtL011G12	IRS1_RAT	Insulin receptor substrate 1 (IRS-1) (pp185)	32.1	9.00E-17
OtL012D01	FBpp0081288	puckered	39.7	1.00E-28
OtL012F02	Q1HEQ6_TRICA	Hedgehog (Fragment)	43.7	7.00E-40
OtL015G10	FBpp0074770	pipe	56.7	4.00E-48
OtL017B11	Q3LFR2_BOMMO	Ecdysone 20-hydroxylase (EC 1149922)	47.2	3.00E-42
OtL017F12	FBpp0081448	hyrax	67.4	1.00E-81
OtL017H05	FBpp0086896	bicaudal	69.3	7.00E-46
OtL017H12	FBpp0087596	Wnt oncogene analog 2	51.6	5.00E-52
OtL019E08	Q16PS8_AEDAE	Mago nashi, putative	95.2	7.00E-78
OtL020B06	Q17DN9_AEDAE	Enhancer of split protein, putative	71.5	4.00E-43
OtL020E02	FBpp0071427	Autophagy-specific gene 8a	93.2	8.00E-60
OtL020F12	FBpp0089344	forkhead box, sub-group O	63.7	5.00E-33
OtP001A11	FBpp0089363	bancal	57.7	1.00E-15
OtP001B03	FBpp0073061	disembodied	53.6	3.00E-28
OtP001C01	FBpp0099532	enabled	79.1	3.00E-47
OtP001F04	FBpp0083832	Spatzle-Processing Enzyme	42.9	4.00E-29
OtP003A12	Q2F5M0_BOMMO	Ras-related protein 2	87.1	4.00E-75
OtP004B11	Q8MYD0_APICA	Creb protein (Fragment)	75.8	5.00E-27
OtP005F11	FBpp0082472	bitesize	46.4	2.00E-31
OtP006B04	Q95UR2_TRICA	Homeodomain transcription factor Prothoraxless	82.4	9.00E-54
OtP006B10	FBpp0080555	Bicaudal D	61.9	1.00E-67
OtP007F08	Q967X9_TRICA	Teashirt-like protein	80.3	5.00E-92
OtP008D04	FBpp0081580	Sex comb on midleg	61.8	8.00E-61
OtP008D05	Q95UA8_TRICA	Cephalothorax	77.6	4.00E-51
OtP009C08	Q1RP84_BLAGE	Ecdysone inducible protein 75 isoform B	58.2	2.00E-61
OtP009H01	Q172A2_AEDAE	Staufen	50.2	1.00E-43
OtP010C08	FBpp0074738	absent, small, or homeotic discs 1	47.6	4.00E-45
OtP011F05	Q8T939_TRICA	Ultrabithorax	74.1	1.00E-32
OtP011F12	FBpp0082957	Autophagy-specific gene 8b	36.4	7.00E-16
OtP012A03	Q176U2_AEDAE	Insulin receptor tyrosine kinase substrate	58.2	6.00E-29
OtP012E04	FBpp0071026	cut	93.0	1.00E-40
OtP012H05	FBpp0080662	tailup	63.3	6.00E-25
OtP012H08	PNT2_DROME	ETS-like protein pointed, isoform P2 (D-ETS-2)	46.0	4.00E-39
OtP013E09	FBpp0073311	dishevelled	39.0	6.00E-08
OtP013G10	FBpp0089312	misshapen	89.5	1.00E-126
OtP014A10	Q1KY82_9MYRI	Abdominal-B	96.3	3.00E-07
OtP014B07	FBpp0079676	basket	81.8	6.00E-09
OtP014B10	DLG1_DROME	Discs large 1 tumor suppressor protein	52.1	9.00E-19
OtP015B03	FBpp0074588	gigas	33.2	5.00E-36
OtP015D05	Q9U7D9_LOCMI	RXR	71.8	2.00E-50
OtP015H03	FBpp0099504	hairy	56.8	6.00E-36
OtP015H08	Q17HJ1_AEDAE	Kuzbanian	89.0	1.00E-119
OtL016D12	Q6B0K6_9CUCU	LIM protein	88.8	4.00E-36
OtP016E12	FBpp0088965	cheerio	78.7	1.00E-108
OtP017C12	FBpp0089115	groucho	68.8	4.00E-47
OtP017F02	FBpp0072535	bab2	56.3	7.00E-26
OtP017F03	FBpp0111762	strawberry notch	86.3	6.00E-34
OtP018C02	FBpp0086622	Additional sex combs	70.0	8.00E-08
OtP018E03	Q17J62_AEDAE	Ras	83.7	9.00E-78
OtP019D06	O02035_TENMO	Ecdysone receptor	72.3	3.00E-40
OtP019F10	ILPR_BRALA	Insulin-like peptide receptor precursor	28.6	2.00E-07
OtP019G07	Q6F2E0_XENTR	Bambi (BMP and activin membrane-bound inhibitor)	36.3	1.00E-09
OtP020A12	FBpp0077963	Ecdysone-induced protein 78C	67.6	2.00E-10

1 = contigs consisting of larval ESTs only; 2 = contigs consisting of pupal ESTs only; and 3 = contigs consisting of larval and pupal ESTs.

### Comparative analysis of the *Onthophagus *transcriptome

While the beetle order is incredibly species rich and diverse, this study represents only the second systematic study of beetle genes [6]. Comparative analyses of gene content in *Tribolium castaneum *revealed that the proportion of universal and insect specific genes is similar to that in other insects [6]. However, the proportion of genes without similarity to other organisms is higher in red flour beetles than in other insects [6]. In order to explore the conservation of *Onthophagus *genes across metazoans, we aligned the 2,781 non-redundant *Onthophagus *sequences to the protein sequences derived from the annotated genomes of *Tribolium castaneum *(NCBI GenBank), *Drosophila melanogaster *(FlyBase), *Caenorhabditis elegans *(Ensembl), human (Ensembl), as well as non-redundant protein dataset (nr) from GenBank (Table 3). We also aligned our translated sequences with combined "invertebrate protein datasets" from NCBI .

**Table 3:** Datasets used in this study.

**Genome**	**Database**	**Version (date uploaded, YYMMDD)**
***Drosophila melanogaster***	FlyBase	5.2 (070725)
***Tribolium castaneum***	NCBI	(060410)
***Homo sapiens***	Ensembl	NCBI 36 release 46 (070803)
***Caenorhabditis elegans***	Ensembl	Wormpep 180 (070819)
**Invertebrate**	NCBI	(080508)
**non-redundant**	NCBI	(080514)

**Figure 1:**
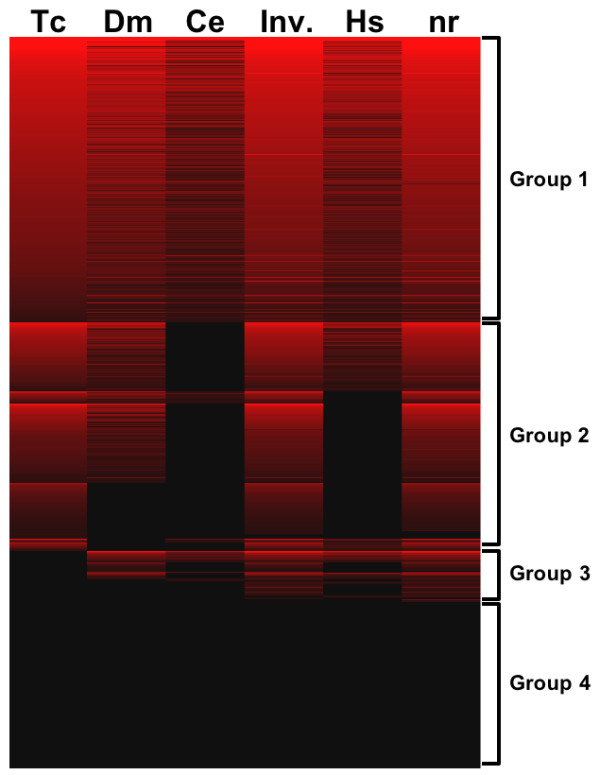
**Overall comparison of *O. taurus *sequences with other protein datasets**. Filtering and clustering analysis of assembled *O. taurus *ESTs based on BLASTx. Shown are bit scores against protein sequences from *Tribolium castaneum *(Tc, NCBI), *Drosophila melanogaster *(Dm, FlyBase), *Caenorhabditis elegans *(Ce, Sanger), invertebrate proteins (inv., NCBI), *Homo sapiens *(Hs, Ensembl), and non-redundant protein dataset (nr, NCBI). Each row represents a single *Onthophagus *sequence, and each column represents sequence matches to proteins from the indicated datasets, where the color intensity is proportional to the bit score (0 = black to 789 = brightest red). The *Onthophagus *sequences are grouped (Groups 1-4) according to the patterns of BLASTx sequence matches with proteins in the various datasets (E-value cut-off = 1 × 10^-5^), and clustered according to the bit scores. There are 1,086, 868, 194, and 633 sequences in groups 1-4, respectively. The complete dataset for this figure is available as Additional file 3.

In order to group the Onthophagus sequences according to patterns of conservation and divergence across these datasets we filtered them for those with BLASTx sequence matches with proteins in the various datasets (E-value < 1 × 10^-5^), and then clustered them according to the bit scores (Figure 1 and Additional file 3). A total of 1,086 non-redundant *Onthophagus *sequences (39%) had sequence matches to proteins in all the datasets searched (Figure 1, Group 1). A further 868 of the non-redundant sequences (31%) had matches to proteins in the *Tribolium *dataset, as well as to proteins in one or more of the other datasets (Figure 1, Group 2). Within group 2 there are two prominent sub-groups. First, there were 300 non-redundant (10.8%) with matches to fly, "invertebrate", and nr proteins, but no matches to worms and humans, and are thus putatively restricted to insects. We manually inspected the matched sequences and confirmed that there are no protein matches from non-insect species. While Gene Ontology annotations of these sequences show no striking enrichment for specific biological processes, they do include proteins with functions specific to insects, such as cuticle proteins (data not shown). Second, there were 212 (7.6%) non-redundant sequences with matches to proteins in either only *Tribolium*, or *Tribolium *and either/both of "invertebrate" and nr proteins. In order to test if these sequences are indeed restricted to beetles we filtered them to meet the following criteria: (i) the *Onthophagus *sequences did not have a sequence match (BLASTx, E-value < 10^-20^) with proteins from non-beetle species in the nr dataset; (ii) the *Tribolium *protein to which the *Onthophagus *sequence had the best alignment did not have a sequence match (BLASTx, E-value < 10^-20^) with proteins from non-beetle species in the nr dataset. In total, 44 of the non-redundant sequences (1.6%) met these criteria and thus are restricted to beetle species among the currently available protein sequence information. This suggests that these genes may have arisen *de novo*, or may be fast evolving, in beetles. A total of 194 translated non-redundant sequences (7%) did not match proteins from *Tribolium*, but did have matches in one or more of flies, worms, humans, invertebrates or nr (Figure 1, group 3). Among these, 43 sequences had matches in *all *other protein datasets, and 33 additional sequences had matches to *Drosophila*, "invertebrate", and nr. Combined, these data raise the possibility that at least some of these 76 sequences may either have been lost from the *Tribolium *lineage, or alternatively, may be conserved but simply not yet annotated in *Tribolium*. Indeed, we found 22 cases where the *Onthophagus *sequences matched against regions of the *Tribolium *genome (BLASTn E-value < 1 × 10^-5^, data not shown) with no gene annotations. These may represent genes that are unannotated in the *Tribolium *genome. Thus, the *Onthophagus *expressed sequences reported here would be useful in refining the annotation of the *Tribolium *genome.

Our analysis also identified 633 of the non-redundant sequences (23.2%) to have "no-hit" (Figure 1 Group 4) to any of the proteomes. This is consistent with the finding that approximately 23% of genes annotated in the *Tribolium *genome lack sequence matches in a wide range of other species [6]. However, our estimate of *Onthophagus *specific sequences is likely to be inflated by (i) sequences that are largely, or entirely, within the UTRs of protein coding transcripts, or (ii) sequences that may be non-coding transcripts. Resolving the question of whether these sequences do in fact represent genes that are unique to *Onthophagus *must await large-scale sequencing of the transcriptomes and/or genomes of *Onthophagus *and related species. However, the observation that 44% of theses sequences include ORFs of greater than 300 nucleotides (data not shown), suggests that at least some of these represent protein-coding genes that have not yet been identified in the species sequenced to date.

### Gene expression profiles in pupal appendage primordia

While our EST analysis identified many genes homologous to interesting *Drosophila *developmental genes, and such an approach to identify candidate genes has been successful in beetles [11,15,19,20], this approach is limited to identifying obvious candidates. Given that *Onthophagus *horns appear to be novel structures invented in beetles, it is highly likely that unexpected, or indeed previously uncharacterized genes may be important in their development. We therefore developed a custom microarray spotted with the 3,756 cDNA clones from which the ESTs were derived (Methods), undertook gene expression profiling of developing horns (early pupal stage) as an unbiased means of identifying such candidates. Since there is evidence that head horns and prothoracic horn are quite distinct structures (not simply serial homologs; [13,16]), we analyzed gene expression in each of these organs separately. Since there is evidence that some, but not all, appendage patterning genes play a role in horn development [15], we included legs in our analysis in order to distinguish similarities and differences between horns and a canonical appendage. Finally, since beetle horns and legs both develop by out-budding of the epithelium, we use non-appendage bearing epithelium (dorsal abdomen) as a common reference sample.

**Figure 2:**
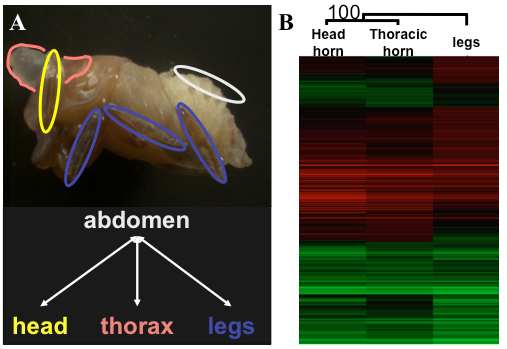
**Experimental design and clustering analysis of the gene expression pattern in *O. taurus *day 1 pupa**. **A**. Microarray experimental design. The pupal tissues used are indicated in the upper panel and the microarray hybridizations are illustrated in the lower panel. Head horn (head) is labeled yellow, thoracic horn (thorax) is labeled pink, legs are labeled blue, and abdominal epithelium (abdomen) is labeled white. **B**. Hierarchical clustering of differentially expressed genes. 1,367 spots were clustered based on their M-values when compared to abdominal epithelium. Each row represents a single spot and each column represents the sample. Relative magnitude of gene expression level is indicated by color brightness; red indicates enriched compared to abdominal epithelium whereas green indicates depleted relative to abdominal epithelium. M-values ranged from -4.85 to 4.12. Bootstrap values were obtained after 5000 trials. Branch lengths represent relative distances between the samples.

The design of the expression profiling experiments included three comparisons, each done with five independent biological replicates (Figure 2A). The complete microarray data are available at GEO , accession number GPL7555). The percentage of array elements that detected signal (where feature intensity > average + 2 SD background intensity) was uniformly high across the four tissues interrogated: an average of 78.5% in head horns, 82.7% in prothoracic horns, 84.3% in legs, and 83.7% in abdominal epithelium. Signal intensities were also reproducible across both technical replicates hybridized on the same microarray (average correlation coefficient = 0.94, n = 2, SD = 0.04), and independent biological replicates hybridized on different microarrays (average correlation coefficient = 0.901, n = 60, SD = 0.062). These data indicate the microarrays were sensitive and the experiments were reproducible. A total of 1,542 of the 3,756 cDNA array elements detected statistically significant differential expression (adjusted p-value < 0.05) in one or more of the three comparisons - head horns, prothoracic horns and legs all compared to abdomen. In order to examine the overall similarities and differences in the patterns of these differentially expressed genes we used two-dimensional hierarchical clustering (175 array elements with some missing data-points were excluded from the clustering). This revealed that expression patterns of head horns, prothoracic horns, and legs are remarkably similar (Figure 2B). Similar results were obtained when the data were collapsed into non-redundant sequences (data not shown). 83% of the array elements (1,135 out of 1,367) detected enriched or depleted expression in all three tissues compared to abdominal epithelium. Despite the high degree of similarity, the expression patterns in head horns and prothoracic horns are still significantly closer to each other than they are to those in legs (reflected in the branch lengths in the sample tree in Figure 2B). 11% of the array elements (150 out of 1,367) detected enriched or depleted expression in both head and prothoracic horns and not legs. Thus, in terms of overall patterns of gene expression, head and prothoracic pupal horn primordia are similar to but distinct from pupal leg primordia.

### Identifying candidate genes based on expression in horn primordia

**Figure 3:**
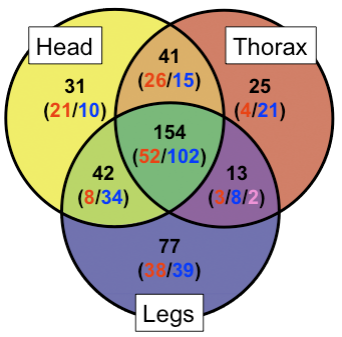
**Expression differences between horn and leg primordia relative to abdominal epithelium**. Categorization of genes that exhibited significantly differential (p value < 0.05 *and *> 2-fold difference). The labels on each category represent the tissue types (head = head horns, thorax = prothoracic horns, and legs = legs). Numbers indicated in the Venn diagram represent the counts of non-redundant sequences in each category. The numbers in parenthesis indicate the counts of sequences that showed enriched or depleted expression relative to abdominal epithelium, where: red = enriched, blue = depleted, and pink = mixed (i.e. enriched in thoracic horns and depleted in legs).

We expected that at least some genes involved in horn development would show differential expression in pupal horn primordia. In order to focus on these genes, we collapsed the data from array elements into non-redundant sequences (contigs and singletons), and then filtered the non-redundant sequences for those that were both statistically differentially expressed and showed at least two-fold changes in either head horns, prothoracic horn or both types of horns. A total of 306 non-redundant sequences met these criteria (adjusted p-value < 0.05 and fold-change > 2); 73 in head horns only, 38 in prothoracic horns only, and 195 in both head and prothoracic horns (Figure 3). These 306 non-redundant sequences included 74 with no BLASTx matches (E-value < 10^-5^) to protein sequences in UniProtKB, *Tribolium castaneum *(NCBI GenBank), *Drosophila melanogaster *(FlyBase), *Caenorhabditis elegans *(Ensembl), human (Ensembl), or non-redundant protein dataset (nr) from GenBank. The remaining 232 were putatively annotated based on matches to proteins from other species (Additional file 4). The candidate genes for the development of beetle horns can be grouped into three conceptual categories (expected, unexpected, and unknown genes).

First, we identified genes whose expression in the context of horn development could be expected given existing insights into the developmental biology of horns, and knowledge about the function of these genes in other organisms. For instance, the Hox gene *Sex combs reduced *(*Scr*) is enriched in the prothoracic horn (19.2 fold) and legs (7.9 fold) relative to abdominal epithelium (Additional file 4). In *Drosophila *and *Tribolium Scr *patterns the identity of the labial and first thoracic segment [21-23]. Preliminary results showed that *Onthophagus Scr *executes similar functions during labial and thoracic development in addition to playing a major role in the regulation of prothoracic horn development (Wasik, Rose, and Moczek, unpublished data).

Secondly, we identified genes that although functionally well characterized in *Drosophila *or elsewhere, would not readily be expected to be expressed in the context of horn development. Genes in this category include the putative ortholog of *Drosophila doublesex *(*dsx*), enriched more than 2-fold in the head and prothoracic horns relative to abdominal epithelium (Additional file 4). In *Drosophila *the expression of sex-specific DSX isoforms regulate somatic sex-determination sexually dimorphic differentiation [24,25]. While *Onthophagus *horns are sexually dimorphic, our observation that the putative *dsx *ortholog is expressed preferentially in the *male *horn tissue when compared to *male *abdominal tissue was unexpected. Expression and functional studies are now under way to identify the role of *dsx *in the development and diversification of horns.

Similarly, we found that the putative *Onthophagus *orthologs of *yellow-c, -e, and -f *were enriched more than 2-fold in head and/or prothoracic horns relative to abdominal epithelium (Additional file 4). The functions of yellow family genes are remarkably diverse and include the regulation of pigmentation [26,27], the production of a major component of royal jelly in the honeybee [28] as well as expression of normal male courtship behavior in *Drosophila *[29]. Combined, these observations suggest that yellow genes may be involved in the regulation of a wide array of sex- or caste-specific functions, at least among insects, though it remains to be determined, what, if any, function the gene family may be executing in *Onthophagus *beetles.

Lastly, we identified 74 genes that were significantly differentially expressed in either head horns or prothoracic horns, or both, that lack obvious homology to proteins in any of the datasets used in this study. Of those 74, at least 29 (39%) contained predicted ORFs with longer than 300 nucleotides (100 codons).

## Discussion

Horned beetles, most notably in the genus *Onthophagus*, are increasingly being recognized as an emerging model system in evo-devo and eco-devo studies [13,30-34]. Below we discuss the major findings of our study and their applicability to ongoing and future research efforts in horned beetles and beyond.

### *Onthophagus taurus *expressed sequences as a resource

The expressed sequences and the corresponding cDNAs presented here provide a valuable entry point for studies of gene function in *Onthophagus taurus*. The sequences derived from normalized larval and pupal cDNA libraries had a low level of redundancy. The 3,488 high quality EST sequences from both libraries assembled into 2,781 non-redundant sequences (contigs and singletons). The low level of redundancy resulted in a sample of sequences derived from a wide range of biological functions.

### The *Onthophagus *transcriptome

This study provides a first pass survey of genes found in *Onthophagus*. Prior to this study, *Tribolium castaneum *was the only species of beetle for which comprehensive sequence information was available [6,35]. Comparative analyses indicate that the gene repertoire of *Tribolium *is consistent with the general trends seen across sequenced insects and vertebrates [6,36]. Our estimates of the proportions of *Onthophagus *sequences that are common to other species are consistent with those in *Tribolium *[6]. For instance, we found that 39% of *Onthophagus *sequences had sequence matches to proteins in all the datasets searched, which is consistent with the *Tribolium *genome in which ~35% of genes have orthologs in all species examined [6]. Of particular interest are the 23% of *Onthophagus *sequences that lack orthology (Group 4 in Figure 1) to proteins from six proteomes including the non-redundant dataset which is very close to the corresponding estimate of 23% of annotated *Tribolium *genes [6]. About 40% of these Group 4 *Onthophagus *sequences exhibited appreciable putative open reading frames and thus need to be considered potentially protein-coding. This group of genes likely contains genes unique to, or fast evolving in, *Onthophagus *beetles, and studies are under way to further characterize and analyze the significance of these genes for the evolution, diversification, and radiation of horned beetles.

### From ESTs to candidate genes for the evolutionary biology of beetle horns and horned beetles

Beetle horns and horned beetles are attractive system to address several current frontiers in evolutionary biology. The EST resources and array results presented here provide the first genomic resources to identify candidate genes, pathways, and networks underlying morphological, behavioral, and developmental aspects of the biology of horned beetles, as well as providing insights into their respective evolutionary histories. Below we briefly highlight two broad categories of current research efforts and how they are being advanced by the results presented here.

### The origins of horns

Beetle horns have attracted attention because they lack obvious homology to other appendages or outgrowths in the insects. Horns therefore constitute an evolutionary novelty. Understanding how novel traits arise from pre-existing variation remains one of the most challenging and poorly understood questions in evolutionary biology.

One hypothesis that has been proposed toward explaining the origin of horns is based on the observation that horns share many morphological and developmental features with traditional appendages (e.g. epithelial origin, prepupal growth, dorso-ventral axis formation, or pupal remodeling presumably via programmed cell death; [10]). Furthermore, in several other respects horns are much simpler than legs or mouthparts (e.g. they lack nerves, muscles, or joints). Horns may therefore have evolved via the large-scale co-option of genes ancestrally used to instruct appendage development. Our microarray results suggest that horns and legs are indeed highly similar in gene expression profiles and support the hypothesis that many genes involved in leg formation may also play a role in horn development. Earlier research has begun to implicate a small subset of appendage patterning genes in horn development (*Distal-less, dachshund, extradenticle, homothorax*, [11,13,15]). The results presented here add a substantial list of gene candidates (Additional file 4) that may have mediated the origin of horns via co-option from traditional appendage development.

At the same time, horn-specific transcription profiles also included genes not represented in developing legs, suggesting that horns should not be viewed solely as being simplified appendages. While this fraction of genes was small in comparison, it nevertheless highlights a possible class of genes involved in developmental processes of horn formation that are not represented, or at least not to the same degree, during the development of traditional appendages. If correct, this would suggest that the origin of horns may have been mediated by the co-option of appendage patterning genes alongside integration of genes and pathways unrelated to appendage formation. Clearly, additional contrasts including the sampling of other developmental time points, as well as gene function studies, are needed to establish the general validity of these conclusions.

### The diversification of beetle horns and horned beetles

Beetle horns and horned beetles are attractive study organisms because they permit investigation of the mechanisms underlying phenotypic diversification on many interesting levels. First, species differ in the body region involved in horn expression: horns may extend from the head, prothorax, or both, and while their function as weapons in male combat appears to be conserved across species, recent studies suggest that different horn types may have originated and diversified at least in part independently of one another [13]. Our results support this scenario by identifying a list of genes whose expression differs significantly across horn types such as *yellow-e *(head horns), *tailup *(encodes a LIM-homeodomain protein; prothoracic horns), or *Scr *(prothoracic horns and legs). While the function, if any, of these candidate genes in the context of horn development remains to be explored our results presented here provide an important starting point toward untangling shared, independent, and convergent aspects in the evolution of different horn types across horned beetles.

Substantial diversity in horn expression also exists *within *species in the form of sexual and male dimorphisms. Sexual dimorphisms are brought about via sex-specific regulation of horn expression whereas male dimorphisms are predominantly the product of nutritional differences experienced during larval life (reviewed in [37]). Endocrine factors such as juvenile hormone (JH) are likely to play important roles in the regulation of both types of diversity [38-40]. Furthermore, the same nutritional or hormonal manipulations affect sexual and male dimorphisms differently in different species and populations, suggesting that evolutionary changes in the interplay between endocrine factors, nutrition, and sexual differentiation have contributed to the diversification of horned beetles [40,41]. Our EST resources and microarray results provide an important starting point to begin exploring putative candidate genes that may be associated with sex-specific (such as *doublesex*, *transformer-2 *or members of the *yellow *gene family) or nutrition-dependent (e.g. *foxo*) expression of horns. Moreover, the resources presented here should support the development of experiments towards characterizing sex- and morph-specific transcriptomes in *O. taurus *and closely related species in the genus (Snell-Rood, Cash, Kijimoto, Andrews, Moczek; unpublished data).

## Conclusion

In conclusion, the EST resources and microarray results present here provide a first step toward a systematic analysis of the molecular basis of horn development and diversification in beetles with the potential to inform several major frontiers in evolutionary developmental biology.

## Methods

### cDNA library construction

Adult *Onthophagus taurus *were collected from pastures near Bloomington, IN and reared as described previously [15]. We constructed two separate libraries from larval and pupal stages. For the larval library we dissected heads and thoraces from mid third instar larvae, late third instar larvae, and early and late prepupal stages. For the pupal library tissues included whole individuals one, two, three and four days after pupation. For both libraries we harvested at least two individuals for each stage and sex, and all samples were frozen in liquid nitrogen, immediately transferred to -80°C for storage until RNA extraction. Total RNA was extracted using TRIreagent (Sigma, MO), precipitated with ethanol and stored at -80°C. The normalized cDNA libraries were each constructed from 1 μg of total RNA, using the TRIMMER-DIRECT cDNA normalization kit (Evrogen, Moscow, Russia) for the library normalization, followed by the Creator SMART cDNA library construction kit (Clontech, CA) for cDNA library construction, as described in Zhulidov *et al*. 2004 [42]. We followed the manufacturers protocols with the following modifications and specific conditions: (i) the cycle conditions for the PCR-based double-strand cDNA synthesis were 16 cycles of [95°C for 7 sec, 66°C for 30 sec, and 72°C for 6 min]; (ii) we used 2 μl of cDNA mixture for PCR during cDNA library construction and normalization; and (iii) the conditions for the two step amplification of the normalized cDNA were 18 cycles [95°C for 7 sec, 66°C for 30 sec, and 72°C for 6 min] for the first step, and the second amplification was cycled for 12 cycles using the same conditions. Normalized and amplified cDNA fragments were size-fractionated, digested by *Sfi *I, and ligated with the plasmid vector pDNR-LIB according to manufacturer's instruction. Electro-transformed *E. coli *cells were spread on LB plate containing chloramphenicol (final concentration of 30 μg/ml). The estimated titer of both of the libraries were ~1 × 10^-8^CFU. A total of 3,756 colonies were picked at random. Unless stated otherwise standard molecular procedures were used to execute basic molecular analyses [43].

### EST sequencing

DNA samples were prepared for sequencing using a Beckman Coulter Biomek FX Laboratory Automation Workstation as described in Burr *et al*. 2006 [44]. Each picked clone was incubated overnight at 37°C in 96-well tissue culture plates with 100 μl of SOC medium with chloramphenicol (final concentration of 30 μg/ml), without rotation. 20 μl of the cultured cells were mixed with 80 μl of water and heat-punctured at 95°C for 10 min. Insert DNA was PCR-amplified using cell lysate (10 μl) as template, 0.1 μM M13fw primer (5'-GTG TAA AAC GAC GGC CAG TAG-3'), 0.1 μM M13rev primer (5'-AAA CAG CTA TGA CCA TGT TCA C-3'), 0.2 mM each dNTP, 0.5 U/20 μl reaction Taq polymerase (Bioline, MA), and 1× reaction buffer (Bioline, MA). The reaction was incubated at 95°C for 5 min then 35 cycles of [95°C for 1 min, 54°C for 1 min, and 2 min at 72°C]. The amplified DNA was purified using the Multiscreen-PCR 96-well purification system (Millipore, MA). The purified DNA was subjected to agarose gel electrophoresis against molecular weight standard and visualized using a Kodak 440cf imaging station. Sequencing reactions were performed with the primer pDNRlib30-50 (5'-TAT ACG AAG TTA TCA GTC GAC G-3') and ABI BigDye chemistry and ABI Prism 3730 sequencer (Applied BioSystems, CA).

### EST processing, assembly, and annotation

ESTPiper [17] was used to analyze EST sequences including base calling, data cleaning, *de novo *assembly, and annotation. A total of 3,756 EST sequences were generated in FASTA format with quality scores after base calling. For data cleaning, ESTPiper first removed low quality and vector sequences using LUCY [45] program with the default parameter settings. PolyA/T tails were then trimmed, where within 50 bp searching range from both ends of the sequences, the minimum length of continuous polyA/T region was set to 9 bp and the maximum number of mismatches within the polyA/T region was set to 3. Potentially chimeric clones, which were defined as sequences with at least 30 bp continuous A/T or adaptors occurring in the middle of sequences, were removed. Finally, shorter sequences (< 100 bp) were also removed. A total of 3,488 high quality sequences passed data cleaning procedure. We then performed *de novo *assembly to assemble EST sequences into contigs and singletons. Parameters were set as follows: (i) overlap percent identity cutoff was 95%, (ii) overlap length cutoff was 49, and (iii) maximum number of word matches was 10,000 (this parameter defines the maximum number of matches that the program will consider for a given sequence, and was set high to improve accuracy [46]. For annotation, ESTPiper matched contigs/singletons to UniProt database [47] using BLASTX with an E-value cutoff of 1 × 10^-5 ^and only the top match was taken.

### Microarray printing

We developed the cDNA microarray using all the clones used for the EST analysis (3,756 clones) as well as GAPDH and actin-5c (internal positive controls). Insert DNA was PCR amplified and purified as described above in the *EST sequencing *section. We followed the protocol of Indiana University Drosophila Genomics Resource Center [48] to print microarrays with a minor revision to post-print washes. Purified insert DNA was dried completely, re-dissolved in DGRC spotting solution (1.5 M Betaine in 3 × SSC), and spotted to GAPSII Microarray Slides (Corning) using an OmniGrid 300 printing. The microarray design included 4,320 spots arranged in 48 blocks of 90. A total of 3,756 of these spots were cDNA fragments (each spotted once) and 564 of these consisted of control spots (GAPDH, actin-5c, and spotting buffer only). The gene list and platform description is available at Gene Expression Omnibus  accession number GPL7555. After printing, the microarrays were heated at 85°C for 3 hrs and rinsed with 5 × SSC/0.1%SDS (55°C), water (twice at RT, once at 95°C, and once again at RT) and then centrifuged to dry. All microarrays were kept dry at room temperature until they were used.

### Target RNA preparation, hybridization and obtaining data sets

Tissues were dissected from 20 male *O. taurus *(day 1 pupae) that were collected from our laboratory colony. Dissections and RNA extractions (RNeasy Mini kit, Qiagen, CA) of head horn, prothoracic horn, leg, and abdominal epithelium were performed separately for each animal. Independent biological replicates of RNA samples were created by pooling an equal mass of RNA isolated from the same type of tissue from 4 individuals. For each RNA sample 1 μg of RNA was reverse transcribed using Oligo(dT)-T7 primer (Ambion, TX) and SuperScriptIII reverse transcriptase (Invitrogen, CA), and DNA polymerase and RNase H (Invitrogen, CA) were used for second strand synthesis. Amplified RNA (aRNA) was generated by *in vitro *transcribing the cDNA using the MEGAscript kit (Ambion, TX). The aRNA was directly labeled with Cy3 or Cy5 using the ULS aRNA Fluorescent Labeling Kit (KREATECH, Amsterdam, The Netherlands). Three sets of amplified RNA samples from head horns, prothoracic horns, and legs were labeled with Cy5, while abdominal epithelial tissue samples were labeled with Cy3. The remaining two sets of samples were labeled in the opposite way. After measuring the quantity and labeling efficiency, amplified and labeled RNA samples from test (head horns, prothoracic horns, and legs) samples and abdomen (reference sample) were mixed and hybridized onto arrays. aRNA with 50 pmol dye from the test sample and reference sample were mixed with KREAblock (ULS aRNA Fluorescent Labeling kit) and 2 × enhanced cDNA hybridization buffer (Genisphere, PA), then heated at 80°C for 10 min. Arrays were pre-treated for more than one hour at 55°C in pre-hybridization buffer (5 × SSC, 0.1%SDS, 1% I-block (Applied Biosystems, CA)). Both mixed sample and microarray were kept at 55°C until the hybridization step. Hybridization was performed in a dark humidified chamber at 55°C overnight. The microarray was rinsed in buffer A (2 × SSC/0.2%SDS) at 55°C then incubated in buffer A at 65°C for 10 min. The microarray was transferred to 2 × SSC (room temperature) for 10 min, followed by incubation in 0.2% SSC for 10 min at room temperature. The rinsed microarray was dried by centrifuging at 500rcf for 4 min.

The hybridized microarrays were scanned by GenePix scanner 4200 (Molecular Devices, CA) to obtain raw data sets. After initial quality check of results using OLIN in Bioconducter (Basic Hybridization Analysis, Costello *et al*. 2005, ), differential expression was assessed using Limma [49]. The values for each spot were shown as log_2 _ratios between the two signal intensities (M-values). The microarray data are available at Gene Expression Omnibus , accession number GPL7555.

### Clustering analysis

We performed clustering analysis and support tree construction using TIGR MultiExperiment Viewer of the TM4 system [50]. We performed hierarchical clustering by using Cosine Correlation with average linkage to obtain the cluster and tree.

## Authors' contributions

TK, APM, and JA designed the study. TK developed cDNA libraries and microarrays. TK, JC, ZT and JA analyzed libraries. TK performed microarray experiments. TK, APM, and JA analyzed the microarray results. TK, JC, ZT, APM and JA wrote the paper. All authors read and approved the final manuscript.

## Supplementary Material

Additional file 1 **Summary of BLASTx results of *Onthophagus taurus *ESTs against UniProtKB**. Summary of BLASTx sequence alignments of non-redundant sequences from Ot libraries (refer Results section) against UniProtKB protein dataset. The top BLASTx hits with E-values < 10^-5 ^are shown. Click here for file

Additional file 2 **Summary of BLASTx results of *Onthophagus taurus *ESTs against FlyBase**. Summary of BLASTx sequence alignments of non-redundant sequences from Ot libraries (refer Results section) against FlyBase protein dataset. The top BLASTx hits with the E-values < 10^-5 ^are shown. Click here for file

Additional file 3 **Summary of BLASTx results of *Onthophagus *ESTs against all the datasets used in this study**. Summary of BLASTx sequence alignments of *Onthophagus *ESTs against all the datasets used in this study. The bit score, E-value, and percent identity as well as the gene names from *Tribolium, Drosophila, C. elegans*, invertebrate, human, and nr dataset are shown for of each Ot non-redundant sequences. Click here for file

Additional file 4 **List of genes exhibiting differential expression in horn- or leg primordia compared to abdominal epithelium**. Listed are genes exhibiting significant (p < 0.05) and at least 2-fold difference in expression intensity in primordial head horns and/or prothoracic horns and/or legs. Shown are spot- or contig IDs, the tissue(s) in which differential expression was detected (H: head horns, T: prothoracic horns, L: legs), fold-differences in expression relative to abdominal epithelium (positive values indicate enrichment relative to abdominal epithelium), sequence match descriptions (UniProtKB, FlyBase, *Triboium*, invertebrate, or nr), percent amino acid sequence identity, E-values, and bit score. Click here for file
